# Natural Killer Lysis Receptor (NKLR)/NKLR-Ligand Matching as a Novel Approach for Enhancing Anti-Tumor Activity of Allogeneic NK Cells

**DOI:** 10.1371/journal.pone.0005597

**Published:** 2009-05-19

**Authors:** Gal Markel, Rachel Seidman, Michal J. Besser, Naama Zabari, Rona Ortenberg, Ronnie Shapira, Avraham J. Treves, Ron Loewenthal, Arie Orenstein, Arnon Nagler, Jacob Schachter

**Affiliations:** 1 Ella Institute of Melanoma, Cancer Research Center, Sheba Medical Center, Ramat Gan, Israel; 2 Talpiot Medical Leadership Program, Sheba Medical Center, Ramat Gan, Israel; 3 Department of Clinical Microbiology and Immunology, Sackler School of Medicine, Tel Aviv University, Tel Aviv, Israel; 4 Tissue Typing Laboratory, Sheba Medical Center, Ramat Gan, Israel; 5 Center for Advanced Technologies, Sheba Medical Center, Ramat Gan, Israel; 6 Institute of Hematology, Sheba Medical Center, Ramat Gan, Israel; Centre de Recherche Public de la Santé (CRP-Santé), Luxembourg

## Abstract

**Background:**

NK cells are key players in anti tumor immune response, which can be employed in cell-based therapeutic modalities. One of the suggested ways to amplify their anti tumor effect, especially in the field of stem cell transplantation, is by selecting donor/recipient mismatches in specific HLA, to reduce the inhibitory effect of killer Ig-like receptors (KIRs). Here we suggest an alternative approach for augmentation of anti tumor effect of allogeneic NK cells, which is founded on profile matching of donor NK lysis receptors (NKLR) phenotype with tumor lysis-ligands.

**Methodology/Principal Findings:**

We show that an NKLR-mediated killing directly correlates with the NKLR expression intensity on NK cells. Considerable donor variability in the expression of CD16, NKp46, NKG2D and NKp30 on circulating NK cells, combined with the stability of phenotype in several independently performed tests over two months, indicates that NKLR-guided selection of donors is feasible. As a proof of concept, we show that melanoma cells are dominantly recognized by three NKLRs: NKG2D, NKp30 and NKp44. Notably, the expression of NKp30 on circulating NK cells among metastatic melanoma patients was significantly decreased, which diminishes their ability to kill melanoma cells. *Ex vivo* expansion of NK cells results not only in increased amount of cells but also in a consistently superior and predictable expression of NKG2D, NKp30 and NKp44. Moreover, expanded NK cultures with high expression of NKG2D or NKp30 were mostly derived from the corresponding NKG2D^high^ or NK30^high^ donors. These NK cultures subsequently displayed an improved cytotoxic activity against melanoma in a HLA/KIR-ligand mismatched setup, which was NKLR-dependent, as demonstrated with blocking anti-NKG2D antibodies.

**Conclusions/Significance:**

NKLR/NKLR-ligand matching reproducibly elicits enhanced NK anti-tumor response. Common NKLR recognition patterns of tumors, as demonstrated here in melanoma, would allow implementation of this approach in solid malignancies and potentially in hematological malignancies, either independently or in adjunction to other modalities.

## Introduction

Natural Killer (NK) cells are lymphocytes that belong to the innate immune branch, comprise 5–15% of the peripheral blood lymphocytes and are able to eliminate without prior antigenic stimulation virus-infected and malignantly transformed cells, but to spare normal healthy cells [1]–[2]. NK cells eliminate tumor cells through perforin/granzyme dependent mechanisms, apoptotic mechanism and through secretion of various inflammatory and Th1-promoting cytokines [reviewed in 3]–[4]. NK cells respond to a variety of bioactive agents, including cytokines such as interleukin-2 (IL-2), IL-12, or interferons, by up-regulation of cytolytic, secretory, and/or proliferative functions [5].

Triggering of effector NK cell functions depends on a balance between inhibitory and stimulating signals [reviewed in 1], [4]. Most inhibitory signals are mediated through Killer Ig-like Receptors (KIR) that recognize various alleles of major histocompatibility complex (MHC) class I molecules [6], and it was postulated that all NK cells express at least one receptor that recognizes a self MHC allele, probably to avoid autoreactivity [7]. However, recent evidence show that up to 20% of the peripheral blood NK cells may be KIR-negative, yet self-tolerant, as these cells probably reflect an immature stage [8]–[10]. In addition, MHC class I independent NK inhibitory mechanisms have already been reported [11]–[12]. NK Lysis Receptors (NKLR) are collectively comprised from the family of natural cytotoxicity receptors (NCR) that includes NKp46 [13], NKp44 [14] and NKp30 [15], and from other main killing receptors such as NKG2D [16] and CD16 [17]. Ligands for some NKLRs are found on abnormal cells, such as virus-infected, transformed or stressed cells [4]. Ligands for other NKLRs, such as NKG2D, are not restricted to abnormal cells, but are usually overexpressed under various conditions [18]. Notably, while the cellular ligands for the NCRs are still undefined, several ligands for NKG2D have been identified and include MICA, MICB, ULBP1, ULBP2, ULBP3 and ULBP4 [18]. CD16 is the high affinity FcγRIII receptor [19], but a cellular ligand is yet to be described. So far, the tumor ligands for the NKLRs are still elusive, as only two viral ligands were identified: hemagglutinins of paramyxoviridae viruses are recognized by NKp46 [20] and NKp44 [21], and the CMV tegument protein pp65 that is recognized by NKp30 [22].

The suppression of NK cells by self MHC class I might be a mechanism that enables malignantly transformed cells to evade NK-mediated elimination. Since KIR-ligands on tumors always match the self NK cell KIR repertoire, autolgous NK cells are constantly susceptible to inhibition. This may at least partially explain the past clinical failure of adoptively transferred autologous NK or lymphokine activated killer (LAK) cells to mediate objective rejection of metastatic solid malignancies [23]–[24]. These notions led to the development of the KIR/KIR-ligand mismatch concept to augment anticancer NK-mediated activity [25]–[27]. This concept can be employed only in an allogeneic setting. The use of allogeneic NK cells has shown substantial clinical benefit against acute myeloid leukemia (AML) after haploidentical and partially mismatched unrelated hematopoietic cell transplantation when KIR/KIR-ligand incompatibility existed in the graft-versus-host (GVH) direction [26]. Surprisingly, in contrast to allogeneic T cells, NK cells seem to have an anti GVH effect [26]. A similar approach based on KIR/KIR-ligand mismatching was pre-clinically developed for allogeneic NK adoptive cell transfer (ACT) in solid malignancies [28]. So far, there is still only limited clinical experience with KIR/KIR-ligand mismatching NK ACT in solid malignancies [29]–[30].

The prevalence of malignant melanoma (MM) is continuously rising, effectively almost tripling over the last 30 years [31]. Usually, MM is diagnosed at an early-stage and is completely amenable to primary surgical treatment. However, MM is sometimes characterized by an aggressive course with widespread metastasis and poor prognosis. Immunomodulating drugs, such as IL-2 comprise the mainstay of systemic therapeutic modalities in disseminated MM [32]. Overall, objective clinical benefits are evident in approximately 20% of the patients treated with IL-2, with some of the patients exhibiting complete response and uncommonly even cure [32]. Recently, attention to cell-based therapy in melanoma has been growing again, especially with tumor infiltrating lymphocytes (TIL), due to several encouraging studies reporting on more ∼50% response as a second-line approach [33]–[35]. Nonetheless, several drawbacks to TIL-based therapy, such as the need for surgery to obtain tumor tissue, the long time and high costs required to develop sufficient numbers of TIL, mandates the development of alternative or complementary cell-based approaches.

Here we present a new concept for augmenting anticancer activity of NK cells, which is based on NKLR/NKLR-ligand matching and is completely independent of KIR/KIR-ligand mismatching. The feasibility of NKLR-based selection of donors and the direct correlation with anti tumor function are shown in malignant melanoma as a lead example. This approach may be implemented in solid malignancies and potentially in hematological malignancies, either independently or in adjunction to other modalities.

## Materials and Methods

### Ethics Statement

This study was approved by an Institutional Review Board. All subjects gave their written informed consent according to the Declaration of Helisnki.

### Cells

Primary melanoma cells were derived from surgically removed specimens obtained from melanoma patients as part of an ongoing clinical trial NCT00287131. Cells were grown and maintained as formerly reported [36]–[37]. C1R cells stably transfected either with an empty vector (C1R/Mock) or with the NKG2D-ligand ULBP1 (C1R/ULBP1) were kindly provided by Dr Ofer Mandelboim (Lautenberg Center of Immunology, Hadassah Medical School of Hebrew University, Jerusalem, Israel).

### Antibodies

The following fluorophore-conjugated monoclonal antibodies were used in this study (all from R&D Systems, Minneapolis, MN, USA): anti-CD3-FITC, anti-CD56-PE/Cy5.5, anti-CD16-PE, anti-NKG2D-APC (clone 149810), anti-NKp46-APC (clone 195314), anti-KIR3DL1-APC, anti-KIR2DL1-PE and anti-KIR2DL2-PE. The monoclonal antibodies anti-NKp30-PE and anti-NKp44-PE were purchased from Miltenyi Biotech (Bergisch Gladbach, Germany). PE-conjugated goat anti human F(ab)2 fragments (Jackson Immunoresearch, West Grove, PA USA) were used as secondary reagent for detection of Ig-fusion proteins.

### NKLR-Ig fusion proteins

The cDNA of extracellular portion of various NKLRs was fused in frame to the cDNA of the Fc portion of human Ig. The NKLR-Ig fusion proteins were produced in COS-7 cells and purified on a protein G column as previously described [11]. Proteins were routinely tested in SDS PAGE for degradation. NKLR-Ig fusion proteins were kindly provided by Dr Ofer Mandelboim.

### Flow cytometry

Two hundred thousand cells per well were seeded in 96-U shaped microplates and incubated in 50 µl of PBS/25% human serum (Sigma, Rehovot, Israel) for 10 minutes on ice for blocking. The appropriate antibody mixture diluted in 50 µl of FACS medium (PBS, BSA 0.5%, sodium azide 0.02%) was further added onto the cells and incubated for 30 minutes on ice under dark conditions. Plates were centrifuged at 400 g for 6 minutes in 4°C, supernatant removed, and each well washed with 200 µl of FACS medium.

### Melanoma patients and healthy donors

Peripheral blood was obtained from 40 melanoma patients with clinical evidence of disease at stage IV according to AJCC staging system, or from 30 healthy donors. Peripheral blood lymphocytes were isolated using density gradient centrifugation and deep frozen until use.

### Enrichment of NK cells

Peripheral blood mononuclear cells (PBMCs) of healthy donors were isolated from Leukapheresis or Buffy Coat by ficol density-gradient centrifugation. Only clinically approved reagents were employed, to enable potential compatibility in the future. NK cells were enriched from PBMCs using a CD3^+^ depletion kit (Miltenyibiotec, Bergisch Gladbach, Germany) according to the manufacturer's instructions. In short, CD3^+^ T cells were magnetically labeled using anti-CD3 mAbs conjugated to micro-beads. Depletion of the magnetically labeled cells was performed through two succeeding columns, LS and LD. Isolated cells were washed with PBS. The CD3^+^ T cell contamination was consistently lower than 1% (while using LD column alone resulted in 2–3% of CD3^+^ T cell remnant).

### Expansion and activation of NK cells

To initiate expansion, CD3-depleted cells were re-suspended in one of the analyzed growth mediums: CellGro (Mediatech Inc) or X-VIVO 10 medium (Lonza, Verviers Sprl, Belgium) containing 5% HS (Gemini Bio-Products, West Sacramento, USA; Blood bank, Magen David Adom, Tel-Hashomer, Israel), 1% penicillin-streptomycin (Lonza). Cultures were supplemented with irradiated (5000rad) allogeneic PBMCs derived from two healthy donors as feeder cells and 500 IU/ml IL-2 (Proleukin, Chiron B.V., Amsterdam, Netherlands). Cells were cultured in 5% carbon dioxide-air humidified atmosphere at 37°C. On day 5 of the expansion, half of the medium was replaced by fresh medium. A second round of “feeder cells” was supplemented on day 7. Every 2–3 days total viable cell number was determined and medium was added to attain a final concentration of approximately 0.5–0.8×10^6^cells/ml. Cells were expanded for a total of three weeks, while at different time points throughout the culture total cell counts and NK purity were determined and portions of the culture frozen.

### Cytotoxicity assays

Cytotoxicity assays based on PI co-staining of CFSE-labeled target cells were performed precisely as described [37]. Briefly, cytotoxicity assays were performed by PI costaining of carboxy-fluorescein diacetate succinimidyl ester (CFSE)-labelled target cells; 5×10^6^ target cells were labeled with 2.5 µM CFSE, which strongly labels cells and allows differentiation between target and effector cells. Ten thousand labelled target cells were coincubated in 96 U-shaped microplates with given amounts of effector cells for 5 hr in a humidified 5% CO2 incubator. Killing rate was assessed by the percentage of PI costaining cells out of gated CFSE-labelled cells. In all experiments the percentage of PI-positive CFSE-labelled cells in wells cultured without effectors did not exceed 15%. Blocking of NKG2D or NKp30 was performed by pre-incubation of NK cells with 2 µg/ml of anti NKG2D (R&D Systems, Minneapolis, MN, USA) or with 4 µg/ml of anti NKp30 (BioLegend, San Diego, CA, USA, Clone P30-15), respectively, for 30 minutes prior to incubation with target cells.

### Statistical analysis

Aparametric *t*-test (Mann-Whitney test), aparametric ANOVA (Kruskal-Wallis test) and Spearman's correlation test were performed with GraphPad PRISM® statistical software.

## Results

### NKG2D-mediated killing correlates with NKG2D expression level by NK cells

**Figure 1:**
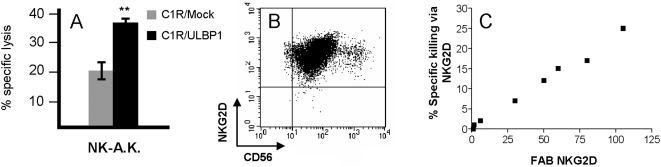
NKG2D-mediated killing directly correlates with the strength of NKG2D expression. Killing rates of C1R cells transfected with an empty vector (C1R/Mock, gray bars) or with the NKG2D-ligand ULBP1 (C1R/ULBP1, black bars) by a representative bulk NK culture NK-A.K. Effector-to-target ratio was 5∶1. ** indicates P value<0.01. (B) NKG2D staining phenotype on a representative bulk NK culture NK-A.K. (C) Correlation between NKG2D expression (X-axis) and percent specific killing via NKG2D (Y-axis). FAB stands for fold above background, which is the ratio between specific and background staining intensity. The percent specific killing via NKG2D is the difference between killing of C1R/ULBP1 to C1R/Mock by the effector cells. Each black square represents the mean values of a different NK bulk culture tested three times. Correlation was tested statistically with Spearman's correlation.

The contribution of an individual NKLR can be directly tested in a cell system based on parental cells devoid of the appropriate lysis ligand, and stable transfectants in which the ligand is expressed ectopically. C1R cells were stably transfected either with one of the known cellular ligands for NKG2D, ULBP1 (C1R/ULBP1), or with an empty vector (C1R/Mock). Both cells were concomitantly tested in killing assays with various primary bulk NK cells. The difference between killing of C1R/ULBP1 and of C1R/Mock reflects the specific killing activity mediated by NKG2D. For example, 20% killing of C1R/Mock, as compared to 37% killing of C1R/ULBP1 (P value<0.01), were observed with primary bulk NK culture NK-A.K. (Figure 1A). The NKG2D expression profile on NK-A.K. is presented on Figure 1B. This implies that the specific killing activity mediated directly through NKG2D was 17%. Expression intensity of NKG2D on NK cells is expressed as fold above background (FAB), which is determined by the ratio between specific staining and background staining intensity. Next, eight different primary bulk NK cultures derived from healthy donors that varied in the expression intensity of NKG2D, were tested in killing assays using the C1R cell system. A remarkable, statistically significant, direct correlation (Spearman's r = 1, p value<0.0001) was observed between NKG2D expression and the magnitude of killing mediated through NKG2D, determined as indicated above (Figure 1C). These results clearly show that killing efficiency is directly affected by the expression intensity of an NKLR, provided that the appropriate ligand is present. Although it is impossible to perform such assays with other NKLRs as the cellular ligands are still mostly undefined, it is conceivable that a similar rule applies as well.

### Significant variability in NKLR expression by NK cells in healthy donors

**Figure 2:**
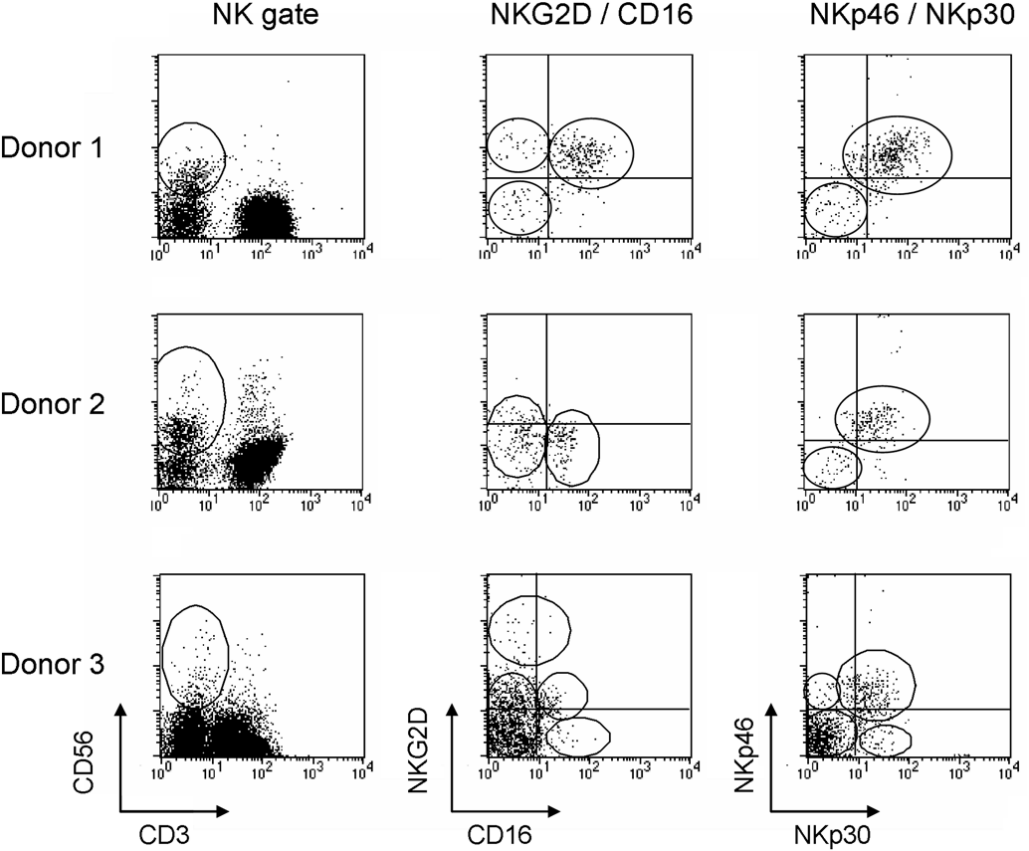
NKLR profile analysis on gated NK cells. Figure shows the NKLR analysis of 3 different donors, as indicated in the left. NK cells were defined as CD56^+^CD3^−^ cells, as depicted in the left vertical panel. Gated NK cells were further co-stained for NKG2D and CD16 (central vertical panel) or for NKp46 and NKp30 (right vertical panel). Circles highlight various subpopulations.

In order to test whether a considerable variability exists in NKLR expression, we have analyzed the expression profile of CD16, NKp30, NKp46 and NKG2D in 20 healthy donors. Analysis was performed on gated NK cells, defined as CD3^−^/CD56^+^ cells (Figure 2, left panels). Various expression patterns were noted, for example; Donor 1, in which ∼90% of the NK cells were positive for CD16, NKG2D, NKp30 and NKp46, yet small subpopulations that lacked one or more of the receptors were clearly observed (Figure 2, upper panels); Donor 2, in which a low expression of NKG2D and CD16 (<50%) was observed, despite a high expression of NKp30 and NKp46 (>90%) (Figure 2, middle panels); Donor 3, in which ∼50% of the NK cells were NKG2D positive and most were CD16 negative. Approximately 35% co-expressed NKp30 and NKp46, while ∼60% did not express either one. Small subpopulations, which expressed only one of the receptors, were detected (Figure 2, lower panels). Staining for NKp44 was negative (data not shown), as this receptor is up-regulated only following activation [11].

**Figure 3:**
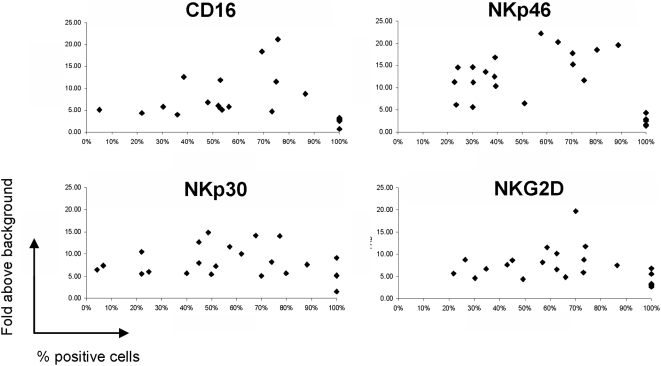
Inter-donor variability in NKLR expression phenotype. Each NKLR was tested on gated NK cells and is presented in a different panel, as indicated. Each dot represents a different healthy donor (N = 20).

The receptor expression profile of 20 healthy donors, including both the percentage of expression of each individual receptor, and its magnitude of expression, were analyzed (Figure 3). Considerable donor variability was clearly evident in both parameters among all NKLRs tested (Figure 3). These results provide at least a partial explanation for the different reactivity usually displayed by NK cells derived from different donors.

### Kinetics of NKLR expression profile on NK cells

**Figure 4:**
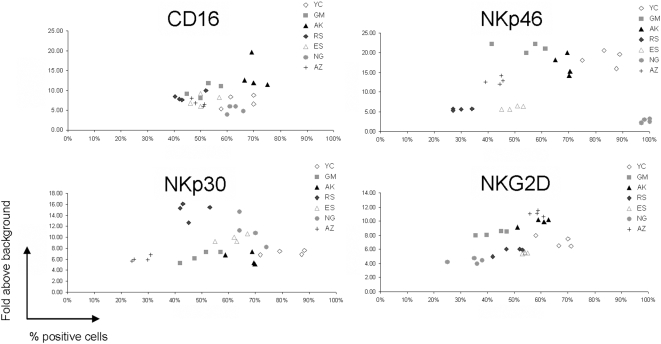
NKLR expression phenotype is stable over time. Each NKLR was tested on gated NK cells and is presented in a different panel, as indicated. Each shape represents a different healthy donor (N = 7).

Periodic fluctuations in NKLR expression in a certain range could account for the differences between healthy donors observed above. We have therefore analyzed NKLR expression profiles in seven different healthy donors longitudinally. Four serial blood samples were obtained from each healthy donor over a period of 2 months (roughly every 2–3 weeks). Peripheral blood lymphocytes were purified on a density gradient from all samples and frozen. On each experiment, all of the serial samples from a given donor were thawed and analyzed in the same experiment. The expression profiles of all NKLRs tested were relatively stable over 2-month period (Figure 4). Only minor variations could be occasionally observed. Nevertheless, the major characteristic differences between some of the donors were clearly maintained over time, for example, low NKp30 expression exerted by donor AZ was stable and remained very distinct from either donor AK (displayed high FAB) or donor YC (displayed high percentage) (Figure 4). These results indicate that although NKLR expression profile on NK cells may vary considerably among different donors, it is generally stable over time in each healthy donor.

### Melanoma cells are recognized by certain NKLRs

**Figure 5:**
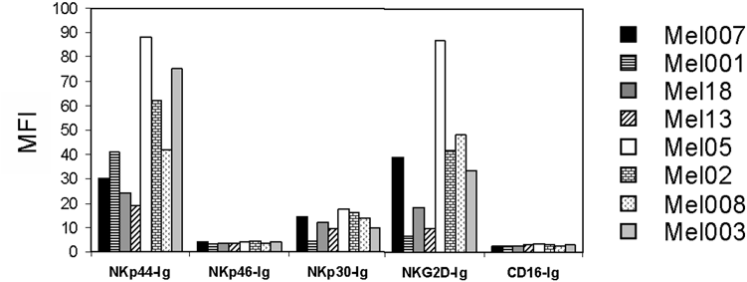
Pattern of NKLR-ligands on primary melanoma cells. Figure shows the median fluorescence intensity (MFI) of the binding of various NKLR-Ig fusion proteins to primary melanoma cells, as indicated in the figure. Figure shows a representative experiment.

The melanoma staining profiles by five different NKLRs, including NKp44, NKp46, NKp30, NKG2D and CD16, were tested. The cellular ligands of these receptors are still unknown, except for NKG2D. Therefore, recombinant chimeric receptors fused to the constant portion of human Ig were generated as described in “Materials and Methods”. Eight different primary melanoma cell cultures were tested, all derived from metastatic lesions. A clear recognition pattern by NKp44-Ig, NKp30-Ig and NKG2D-Ig was observed (Figure 5), as opposed to little or no binding of NKp46-Ig and CD16-Ig (Figure 5). Specifically, NKp44-Ig bound all eight melanoma lines, and NKG2D-Ig as well as NKp30-Ig bound the same seven out of eight melanoma lines. The binding of NKp30-Ig was generally lower than NKp44-Ig and NKG2D-Ig (Figure 4), but this does not necessarily attest for a lesser role of NKp30. No binding of NKp46-Ig or CD16-Ig could be observed to any of the melanoma lines tested (Figure 5). These results suggest that NK-mediated control of melanoma cells is mediated mainly through certain receptors.

### Abnormal NKLR expression by peripheral blood NK cells among melanoma patients

**Figure 6:**
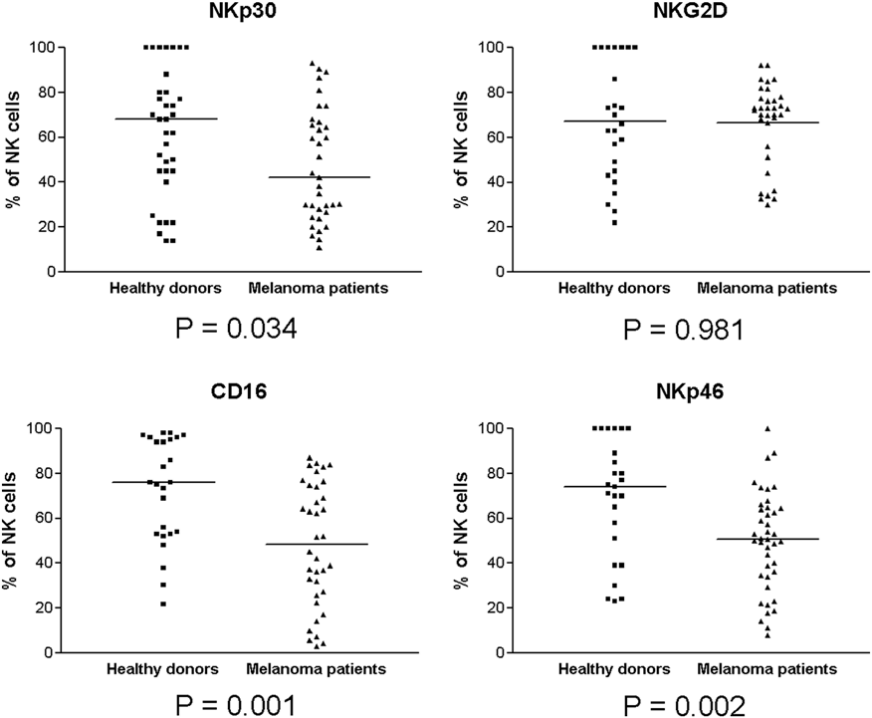
NK NKLR expression profile in healthy donors vs. melanoma patients. The expression profile of each NKLR is presented in an independent panel, as indicated. Each dot represents either a healthy donor or a melanoma patient. Blood specimens were collected from 30 healthy donors and 40 stage IV melanoma patients with clinical evidence of disease. Y-axis denotes the percent of NKLR-positive cells out of gated NK cell. Horizontal lines represent median values. P value under each panel was calculated with an aparametric t-test and represents the statistical significance of the difference between median values.

The expression profiles of NKp30, NKp46, CD16 and NKG2D were tested on peripheral blood, unmanipulated, NK cells. Blood specimens were collected from 30 healthy donors and 40 stage IV melanoma patients with clinical evidence of disease. A significantly lower percentage of NK cells expressed NKp30, CD16 and NKp46 in stage IV melanoma patients, as compared to healthy donors (Figure 6). The potential clinical significance of the NKp30 down-regulation is highlighted by its importance in recognizing melanoma cells, as suggested by the results described in Figure 5. On the other hand, CD16 was substantially down-regulated in melanoma patients (Figure 6) and did not directly bind to melanoma cells (Figure 5). CD16 functions alternatively as a main Fc receptor mediating antibody dependent cell cytotoxicity. In addition, despite the importance of NKG2D in recognizing melanoma cells (Figure 5), no substantial differences were observed between stage IV melanoma patients and healthy donors (Figure 6). Little or no expression of NKp44 was observed, probably as it is induced only following activation (data not shown). Collectively, these observations imply that down-regulated NKLR expression, particularly of NKp30, by NK cells in melanoma patients may affect their ability to recognize and eliminate tumor cells.

### Consistent changes in NKLR expression during NK ex vivo expansion

**Figure 7:**
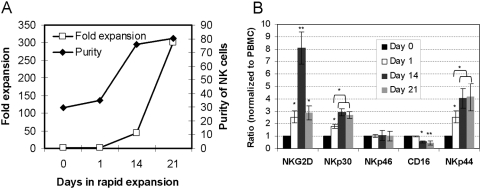
Expression of NKLRs on NK cells during activation and expansion. (A) Ex vivo expansion of peripheral blood NK cells. Figure shows the average results of NK cultures derived from different donors (N = 7). % NK purity was determined by double staining for CD3 and CD56 in flow cytometry. CD3-depleted cells (Day 0) were either activated overnight with 500 IU/ml IL-2 (Day 1) or underwent rapid expansion for 14 (Day 14) or 21 days (Day 21). Fold expansion was calculated relatively to the amount of NK cells in Day 0. (B) NKLR expression during *ex vivo* expansion. All NK cultures were independently stained for the indicated NKLRs. Expression of NKLRs in each donor was normalized according to the corresponding expression on gated NK cells by the un-manipulated peripheral blood mononuclear cells (PBMC). Figure shows the average of normalized results on all healthy donors tested (N = 7). Error bars represent standard error. * denotes a statistical significance of P value<0.05. ** denotes a statistical significance of P value<0.01.

Most forms of adoptive cell immunotherapy require a large number of transferred cells [38]. The long term proliferative potential of NK cells *ex vivo* and the variations in NKLR expression were thus studied. Adhering to clinically approved protocols, CD3-positive cells were depleted from peripheral blood lymphocytes derived from seven healthy donors. This resulted in enrichment of NK cells to around 30% (Figure 7A), as confirmed by CD3/CD56 double staining (data not shown). Some of the CD3-depleted cells (Day 0) were designated for overnight activation with 500 IU/ml IL-2 (Day 1), while others were rapidly expanded as detailed in “Materials and Methods”. Overnight activation with IL-2 had little effect on both purity and fold expansion (Figure 7A). Rapid expansion was stopped after 14 or 21 days. The average fold of cell expansion was 44 and 300, respectively. The average NK purity rates were 76% and 80%, respectively (Figure 7A). Most of the remaining cells were T cells, mainly CD8^+^ cells (data not shown). More than 97% of the cells were viable and were not stained by Propidium Iodine at any time point (data not shown). Thus, NK cells can be effectively and expanded in clinically compatible conditions, with an obvious numerical benefit over simple overnight activation with IL-2.

The expression intensities of NKG2D, NKp30, NKp46, CD16 and NKp44 were monitored on NK cells during activation and expansion. Due to the variability in NKLR profiles among different healthy donors (Figure 3), NKLR expression had to be normalized. Normalization was performed by dividing the fold above background (FAB) of certain NKLR on expanded culture by the FAB of the same NKLR on the corresponding unactivated NK cells gated from un-manipulated peripheral blood lymphocytes. FAB calculations are based on the median fluorescence intensity (MFI) parameter. The CD3-depletion process did not affect the NKLR expression profiles of unactivated NK cells (Figure 7B). A moderate, yet statistically significant enhancement in the expression of NKG2D, NKp30 and NKp44 was observed following overnight activation with IL-2 (P value<0.05). There were no changes in the expression of NKp46 and CD16 (Figure 7B). During rapid expansion process, the various receptors were differentially regulated. NKG2D was strongly induced by day 14 (P value<0.01), but expression subsided steeply by day 21, although it remained higher than unactivated NK cells (P value<0.05). The enhanced expression of NKp30 and NKp44 was further mildly but significantly increased during rapid expansion (Figure 7B, P value<0.05). The expression of CD16 was significantly decreased during rapid expansion by day 14 (P value<0.05), and even more by day 21 (P value<0.01) (Figure 7B). The expression of NKp46 remained unchanged throughout the expansion process (Figure 7B). These results clearly show that overnight activation of NK cells with IL-2 manifests in enhanced expression of various NKLRs. However, strong and prolonged activation during rapid expansion further significantly affects NKLR expression profile, in a predictable and consistent manner. The rapid expansion process mainly affected the NKLRs identified as crucial in recognition of melanoma cells (Figure 5). These combined observations indicate that rapid expansion may be superior to overnight activation with IL-2 by inducing the expression of relevant NKLRs.

### Association between NKLR expression prior to activation and post ex vivo expansion

**Figure 8:**
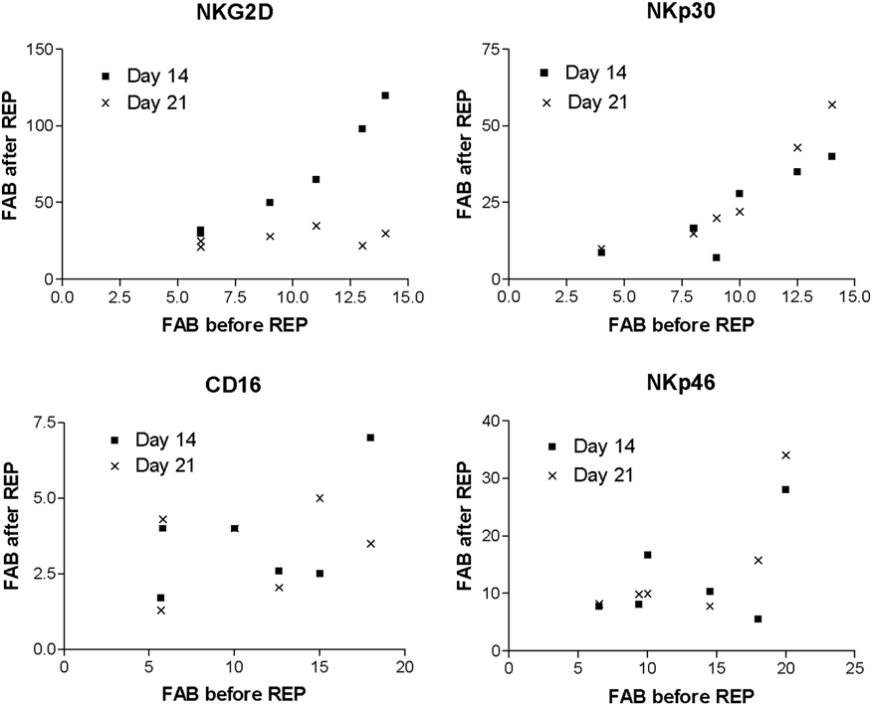
Correlation between NKLR expression prior to and after rapid expansion. Each NKLR was tested on gated NK cells and is presented in a different panel, as indicated. NK cultures from different donors (N = 7) were tested at day 14 (black squares) and at day 21 (X's) of rapid expansion protocol (REP). The NKLR expression prior to rapid expansion was tested on un-manipulated peripheral blood NK cells. Each dot represents the average of at least three staining experiments. FAB - fold above background.

NKLR expression profile varied among healthy donors (Figure 3) and could be predictably manipulated during rapid expansion process (Figure 7). We next tested whether there is an association between initial NKLR expression profile on unactivated NK cells and NKLR expression following *ex vivo* expression. A striking direct association (Spearman's r = 0.97, P value<0.01) was observed between initial expression of NKG2D and its expression at day 14 of *ex vivo* expansion (Figure 8). Higher expression levels of NKG2D were expressed following 14 days of *ex vivo* expansion on NK cells that initially expressed higher levels of NKG2D prior to activation. NKG2D expression was markedly and consistently lower at day 21, and had no correlation with initial NKG2D expression (Figure 8). Initial expression of NKp30 was directly associated with NKp30 expression at both days 14 and 21 (r = 0.82, P value = 0.05 and r = 1, P value<0.01) (Figure 8). In contrast, there was no association between the initial expression level of NKp46 or CD16 with their expression following rapid expansion process (Figure 8). These results show that for some NKLRs, the induction following *ex vivo* stimulation is predictable and depends on the basal expression intensity (donor-specific characteristics) and on length of expansion process (culture-specific characteristics).

### NKLR expression profile correlates with killing efficiency of allogeneic melanoma cells

**Figure 9:**
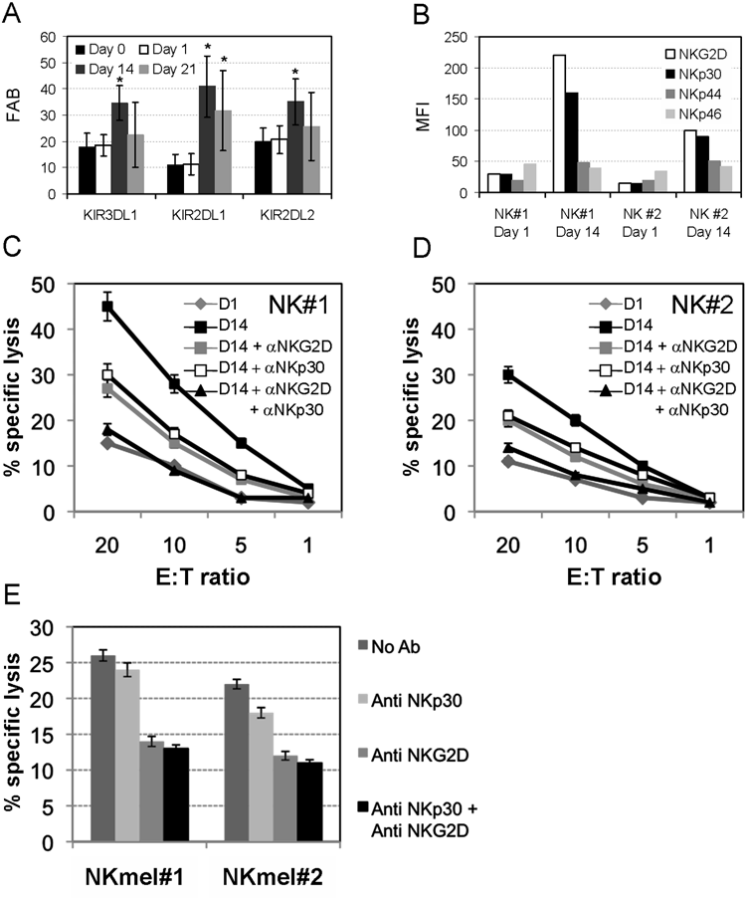
Enhanced expression of NKLRs correlates with improved cytotoxicity against melanoma cells. (A) KIR expression during ex vivo expansion. All NK cultures were independently stained for the indicated KIRs. Expression of KIRs in each donor was normalized according to the corresponding expression on gated NK cells by the un-manipulated peripheral blood mononuclear cells (PBMC). Figure shows the average of normalized results on all healthy donors tested (N = 7). Error bars represent standard error. * denotes a statistical significance of P value<0.05. (B) The expression of each NKLR was tested on two representative bulk NK cultures on day 1 and day 14 of ex vivo expansion process, as indicated in the figure. Expression is presented as median fluorescence intensity (MFI). (C) NK#1 and (D) NK#2 cultures were tested in killing assays against Mel008 cells in various effector-to-target (E∶T) ratios. NKG2D or/and NKp30 on NK cultures #1 and #2 at day 14 were blocked by pre-incubation with mAb at a concentration of 2 µg/ml or 4 µg/ml, respectively. Figure shows a representative experiment. (E) NK cells with low expression of NKp30 were derived from melanoma patients (NKmel#1 and NKmel#2) and tested in killing assays against Mel008 cells in an E∶T of 20∶1. NKG2D or/and NKp30 were blocked as described above. Figure shows a representative experiment.

NK cells were derived and cultured from healthy donors. Cells were analyzed for NKLR expression profiles and killing activity against the primary melanoma cell culture Mel008. Mel008 cells were positively stained by the NKp44-Ig, NKp30-Ig and NKG2D-Ig fusion proteins (Figure 5). The relative contribution of NKLRs to the killing of Mel008 between different NK donors could be compared in HLA-KIR matched or mismatched setups. However, the expression of KIR varies significantly among different donors [39]. This would make the interpretation of NKLR contribution impossible in the matched HLA-KIR setup, as the overall killing would be skewed by the varying inhibitory signals. In addition, the expression of various KIRs alters during *ex vivo* expansion (Figure 9A). We show that the expression of KIR3DL1, KIR2DL1 and KIR2DL2 is enhanced during expansion, while KIR3DL1 and KIR2DL2 tend to return to basal levels after 21 days in culture (Figure 9A). This observation renders the analysis of NKLR contribution in the same NK culture at different time points during expansion problematic as well.

On the other hand, in the HLA-KIR mismatched setup, the inhibitory effect by KIRs is reduced to minimum [25]–[28]. The HLA of Mel008 cells was fully genotyped, and the cells were found to be homozygous for HLA-C from the C1 KIR-Ligand subgroup. Further, they did not express other HLA –A or –B alleles that are recognized by known KIRs (Table 1). Two healthy donors that were homozygous for HLA-C from the C2 KIR-Ligand subgroup were identified, and were thus completely mismatched with the Mel008 cells (Table 1). Since the melanoma cells did not express KIR-ligands from the HLA-A or –B alleles, the fact that the NK donors were positive for these alleles was irrelevant. NK cells were derived from these donors, expanded *ex vivo* and stained for the expression of NKG2D, NKp30, NKp44 and NKp46. The expression of NKG2D and NKp30 on NK cells was higher in Donor #1 than in Donor #2 before, mainly after *ex vivo* expansion (Figure 9B). Following *ex vivo* expansion, the expression of NKp44 was similarly induced in both donors, and the expression of NKp46 was not significantly affected (Figure 9B). Concurring with the differences in expression of NKG2D and NKp30, NK#1 cells displayed higher cytotoxic activity than NK#2 cells in all effector-to-target ratios tested (Figure 9C–D). At day 1 there was only a small difference in the killing activity of NK#1 and NK#2, which fitted the small absolute difference in NKG2D and NKp30 expression (Figure 9B). NK cells from both donors at day 14 of *ex vivo* expansion displayed higher cytotoxic activities than in day 1 (Figure 9C–D). The differences between NK cultures at day 1 and at day 14 may also be accounted to the enhanced NKp44 (Figure 9B). The involvement of NKG2D and of NKp30 in the recognition and elimination of Mel008 cells was evident by the substantial reduction in cytotoxic activity in the presence of a blocking anti-NKG2D mAb or a blocking anti-NKp30 mAb. Blocking of both NKG2D and of NKp30 further decreased the elimination of Mel008 additively, but not abolish it completely (Figure 9C–D). Residual killing activity of Mel008 cells is probably mediated via other NKLRs such as NKp44. Similar results were observed with additional NK cultures tested (data not shown). In conclusion, higher killing efficiency of NK cell derived from certain donors, as well as at various time points of expansion process, correlates with the expression intensity of the relevant NKLRs.

**Table 1:** HLA haplotype of melanoma and NK donors.

	HLA-A			HLA-B			HLA-C			
	Allele1	Allele2	KIR-Ligand	Allele1	Allele2	KIR-Ligand	Allele1	Allele2	KIR-Ligand	
									C1	C2
Mel008	A*02	A*02	NO	B*15	B*15	NO	Cw*3	Cw*3	YES	NO
NK#1	A*01	A*03	YES	B*37	B*57	YES	Cw*6	Cw*6	NO	YES
NK#2	A*30	A*31	NO	B*13	B*35	YES	Cw*4	Cw*6	NO	YES
NKmel#1	A*01	A*01	NO	B*35	B*35	NO	Cw*4	Cw*4	NO	YES
NKmel#2	A*01	A*24	NO	B*35	B*35	YES	Cw*6	Cw*6	NO	YES

Table shows the full high resolution HLA typing of Mel008 cells, as well as the healthy NK donors (NK#1 and NK#2) and melanoma NK donors (NKmel#1 and NKmel#2).

Finally, NK cells with low NKp30 expression were derived from 2 melanoma donors (Figure 6) and tested against HLA-KIR mismatched Mel008 cells (NKmel#1 and NKmel#2, Table 1). An intermediate level of basal killing activity was observed. A minor effect was observed in the presence of the anti NKp30 blocking antibody, as compared to a clear blocking effect observed with the anti NKG2D mAb (Figure 9E). The combination of the two blocking antibodies did not yield an additive effect (Figure 9E), as compared to the effect on healthy donor NK cells (Figure 9C–D). These results indicate that the decreased expression of NKp30 on the surface of NK cells in melanoma patients may culminate in partially impaired response against the melanoma cells.

## Discussion

Cellular immunotherapy holds a great promise in both hematological and solid malignancies. Clinical attempts to employ the anti-neoplastic potential of autologous NK cells were conducted in the past, without achieving substantial beneficial results [23]–[24]. Although failure may emanate from autologous KIR/KIR-ligand interactions, our present study also suggests that at least in melanoma, autologous NK cells have decreased expression of stimulating NKLRs (Figure 6). Several years ago the theory of KIR/KIR-ligand mismatching was developed for allogeneic setting in order to decrease the inhibitory interactions and thus facilitate anti-neoplastic effect. This hypothesis was successfully implemented in AML [26]–[27], but failed in acute lymphocytic leukemia (ALL) patients [26]. The lack of compelling results of this approach in ALL could be explained by paucity of NKLR-ligands on ALL cells [26].

In this study we show that NKLR/NKLR-ligand matching directly affects and improves NK killing activity. A direct correlation between the magnitude of NKG2D-mediated killing and the magnitude of NKG2D expression by NK cells was observed (Figure 1). It is expected that a similar rule applies for other NKLRs. Efficient matching of NKLRs to cancer cells relies on the hypothesis that each cancer histotype is recognized mainly by certain NKLRs. We show here, for example, that melanoma cells derived from different patients are predominantly recognized by three NKLRs: NKG2D, NKp30 and NKp44 (Figure 5). These receptors are therefore presumed to have a dominant role in NK cell response to melanoma, and should therefore be the focus of NKLR/NKLR-ligand matching efforts in this case. Other cancer histotypes may be recognized by other NKLRs. Indeed, killing assays in a HLA-KIR mismatched setup, which neutralizes confounding inhibitory effects, show that NK cells with higher expression of the dominant NKLRs display enhanced cytotoxic activity (Figure 9). It is currently accepted that killing activity reflects the overall activating and inhibiting signals [1], and thus NKLR/NKLR-Ligand matching will enhance NK stimulating signals in the presence of other inhibitory signals.

The specific involvement and additive effect of NKG2D and NKp30 were evident by the partial abolishment of cytotoxic activity in the presence of blocking monoclonal antibodies (Figure 9). Residual killing activity is probably enabled via NKp44. Further, we found that NKp30 is expressed in significantly lower levels among NK cells of metastatic melanoma patients as compared to healthy donors (Figure 6), and consequently that blocking of NKp30 did not alter the anti melanoma function of these NK cells (Figure 9E). It is unclear whether this observation is a melanoma-driven effect (active suppression of NKp30 expression), or whether individuals with a natural lower expression of NKp30 are more susceptible to the development of melanoma (a selection effect).

Allogeneic NK cells could be employed for the treatment of metastatic solid malignancies in an adoptive transfer approach. Allogeneic NK ACT approach have several important advantages: 1) NK cells can be easily obtained from the peripheral blood; 2) NK cells do not require specific antigenic stimulation or selection; 3) They require significantly lower *in vivo* concentrations of IL-2 for maintenance [30], which might have a direct impact on treatment tolerability and toxicity [40]; 4) Allogeneic NK cells probably cannot eliminate normal recipient cells, because they do not express significant levels of the NKLR-ligands [41], and hence are not involved in mediating GVH effects, but rather protect from them [26]; 5) NK cells can be modularly combined with other therapeutic modalities such as antibodies or other autologous cellular products; 6) Allogeneic sources increases the pool of potential donors. Following the success in AML, the potential of KIR/KIR-ligand mismatching was preliminarily studied in solid malignancies. Preclinical studies have demonstrated the enhanced *in vitro* efficacy of KIR/KIR-ligand mismatching against melanoma and renal cell carcinoma (RCC) cells [28]. Clinical studies were performed with peripheral blood NK cells from haploidentical donors that were activated *ex vivo* with IL-2 overnight in AML, melanoma and RCC patients [30]. These studies demonstrated the safety of this modality, as no severe adverse or toxic effects were recorded [30]. Our present results show a significant enhancement in both NKLR expression profile and anti-neoplastic activity of NK cells activated overnight with IL-2 compared with NK cells that underwent *ex vivo* expansion, with the latter being clearly superior (Figures 7, 9). Therefore, these data provide a motivation for *ex vivo* expansion to significantly improve the clinical results with allogeneic NK ACT.

This report, however, further suggests that certain healthy donors with maximal expression of certain NKLRs could theoretically yield NK cells with improved activity against certain cancer cells. It was reported that all mature peripheral blood NK cells express NKG2D and NKp46, and the majority of NK cells express other NKLRs such as NKp30 and CD16 as well [1]–[5]. In contrast, our analysis shows that there is a considerable variability among healthy donors in percentage of receptor-positive NK cells and in magnitude of expression (Figures 2, 3). In some cases, the majority of the NK cells indeed co-express all NKLRs tested, while in other cases, we detected various NK subpopulations that were negative for one or more of the NKLRs (Figure 2). Weak or no expression of NKLRs by peripheral blood NK cells was shown in patients early after bone marrow transplantation, as indicator for immature phenotype [42]. It is not reasonable that the majority of NK cells in the peripheral blood of healthy donors are immature, but as demonstrated with KIR-negative NK cells [8]–[10], their proportion could be significant. Furthermore, it should be noted that in many of the previous studies, primary NK cells were characterized following *in vitro* purification, activation and culture for at least several days. We show that at least the expression of NKp30 and NKG2D is directly induced by *in vitro* activation and culturing (Figure 7B). Other differences may also be attributed to the affinity of different monoclonal antibodies used.

This donor variability would potentially allow selection of optimal donors according to NKLR expression. In the case of melanoma, for example, healthy donors with maximal expression of NKp30, NKp44 and NKG2D would be favored. Since NKp44 is only rarely found in normal peripheral blood, as it is activation-induced [14], maximal expression of NKG2D and NKp30 remains the main criteria for selection of donors. It is possible that other malignant cells would be recognized mainly by a different panel of NKLRs, which would implicate in different selection criteria of optimal NK donors. Thus, although the present study focuses on melanoma, this approach might still fit other malignancies as well, including both hematological and solid origins. Moreover, this approach is not limited by heterozygous expression of KIR-ligands, which is considered as a contraindication in KIR/KIR-ligand mismatching [26]. We show that NKLR expression profile is generally stable and reproducible among healthy donors, at least over two months (Figure 4). Our studies should be expanded and performed on additional healthy donors and over an extended period of time, to fortify these observations. Another important point for future studies is standardization of NKLR expression, for example by normalization according to standard cells, or by employment of a different method such as quantitative real time PCR.

We show that *ex vivo* expansion process has profound, yet generally consistent and predictable, effects on NKLR expression profiles. Indeed, NK cells following 14 or 21 days of *ex vivo* expansion displayed significantly enhanced NKp30, NKp44 and NKG2D expression profiles as compared to overnight activation with IL-2 or fresh cells (Figure 7). In addition, dynamics of NKLR expression patterns over time during *ex vivo* expansion were noted, as evident by the statistically significant differences between NKLR profiles on day 14 and day 21 (Figure 7). Thus, various cultivation conditions might be employed to differentially maximize expression of certain NKLRs of interest. Moreover, initial differences between donors were maintained even following *ex vivo* expansion, e.g. NKG2D^high^ donors yielded NK cells with highest expression of NKG2D (Figure 8), which translates into higher cytotoxic activity (Figure 9). Further investigations would be required for development of NKLR-guided specific cultivation conditions. It should be noted that *ex vivo* expansion significantly enhanced the expression of various inhibitory KIRs as well (Figure 9). This induction may diminish the enhanced NKLR-mediated effect, but an HLA-KIR mismatched setup can alleviate the inhibition, as shown by others [26], [30]. In addition, the NK cultivation protocols employed in this study resulted in more than 20% contamination of T cells, which obviously precludes its clinical use in the present form, due to potential graft versus host disease. Future protocols can be modified, for example: to add an additional T cell depletion round, add an additional NK positive selection round or alter culture conditions.
